# Genomic analysis demonstrates that histologically-defined astroblastomas are molecularly heterogeneous and that tumors with MN1 rearrangement exhibit the most favorable prognosis

**DOI:** 10.1186/s40478-019-0689-3

**Published:** 2019-03-15

**Authors:** Norman L. Lehman, Aisulu Usubalieva, Tong Lin, Sariah J. Allen, Quynh T. Tran, Bret C. Mobley, Roger E. McLendon, Matthew J. Schniederjan, Maria-Magdalena Georgescu, Marta Couce, Mohanpal S. Dulai, Jack M. Raisanen, Mousa Al Abbadi, Cheryl A. Palmer, Eyas M. Hattab, Brent A. Orr

**Affiliations:** 1 Department of Pathology and Laboratory Medicine, University of Louisville, Louisville, KY 40292 USA; 2 Department of Biomedical Informatics, University of Arkansas for Medical Sciences, Little Rock, AR 72205 USA; 3 Department of Biostatistics, St. Jude Children’s Research Hospital, Memphis, TN 38105 USA; 4 Department of Pathology, St. Jude Children’s Research Hospital, 262 Danny Thomas Place, Memphis, TN 38105 USA; 5 Department of Pathology, Vanderbilt University School of Medicine, Nashville, TN 37232-2561 USA; 6 Departments of Pathology, Duke University Medical Center, Durham, NC 27710 USA; 7 Department of Pathology, Emory University, Atlanta, GA 30322 USA; 8 Department of Pathology, Louisiana State University Health Shreveport, Shreveport, LA 71103 USA; 9 Department of Pathology, Case Western Reserve University, Cleveland, OH 44106 USA; 10 Department of Pathology, Beaumont Hospital, Royal Oak, MI 4807 USA; 11 Department of Pathology, The University of Texas Southwestern, Dallas, TX 75390 USA; 12 Department of Pathology, Microbiology and Forensic Medicine, University of Jordan, Amman, Jordan; 13 Department of Pathology, University of Utah, Salt Lake City, UT 84112 USA

## Abstract

Astroblastoma (AB) is a rare CNS tumor demonstrating abundant *astroblastomatous pseudorosettes.* Its molecular features have not been comprehensively studied and its status as a tumor entity is controversial. We analyzed a cohort of 27 histologically-defined ABs using DNA methylation profiling, copy number analysis, FISH and site-directed sequencing. Most cases demonstrated mutually exclusive *MN1* rearrangements (*n* = 10) or *BRAF*^*V600E*^ mutations (*n* = 7). Two additional cases harbored *RELA* rearrangements. Other cases lacked these specific genetic alterations (*n* = 8). By DNA methylation profiling, tumors with *MN1* or *RELA* rearrangement clustered with high-grade neuroepithelial tumor with MN1 alteration (HGNET-MN1) and *RELA*-fusion ependymoma, respectively. In contrast, *BRAF*^*V600E*^-mutant tumors grouped with pleomorphic xanthoastrocytoma (PXA). Six additional tumors clustered with either supratentorial pilocytic astrocytoma and ganglioglioma (LGG-PA/GG-ST), normal or reactive cerebrum, or with no defined DNA methylation class. While certain histologic features favored one genetic group over another, no group could be reliably distinguished by histopathology alone. Survival analysis between genetic AB subtypes was limited by sample size, but showed that *MN1*-rearranged AB tumors were characterized by better overall survival compared to other genetic subtypes, in fact, significantly better than *BRAF*^*V600E*^-mutant tumors (*P* = 0.013). Our data confirm that histologically-defined ABs are molecularly heterogeneous and do not represent a single entity. They rather encompass several low- to higher-grade glial tumors including neuroepithelial tumors with MN1 rearrangement, PXA-like tumors, RELA ependymomas, and possibly yet uncharacterized lesions. Genetic subtyping of tumors exhibiting AB histology, particularly determination of *MN1* and *BRAF*^*V600E*^ status, is necessary for important prognostic and possible treatment implications.

**Electronic supplementary material:**

The online version of this article (10.1186/s40478-019-0689-3) contains supplementary material, which is available to authorized users.

## Introduction

Astroblastomas (ABs) are rare glial neoplasms characterized by relatively compact growth and a predominantly perivascular tumor cell arrangement [1]. They are mostly superficial cerebral lesions presenting in the first to fourth decades of life, with some studies demonstrating a strong female predominance (reviewed by Aldape and Rosenblum [1]). ABs have not been assigned a specific World Health Organization (WHO) tumor grade. They are usually benign; however, clinically aggressive AB cases have been reported [1, 15].

The ontologic and diagnostic significance of ABs has long been debated. Authors have variably argued that they are variants of diffuse astrocytoma or ependymoma [14]. We recently provided evidence that ABs may be related to pleomorphic xanthoastrocytomas (PXAs), based on overlapping clinicopathologic features and detection of the V600E mutation of the B-Raf serine-threonine kinase (*BRAF*^*V600E*^) in a subset of lesions [15].

MN1 (meningioma [disrupted in balanced translocation] 1) is a transcriptional coregulator important in development and is implicated in the pathogenesis of meningioma and acute myeloid leukemia [9, 16]. A recent study of tumors diagnosed as CNS primitive neuroectodermal tumors (PNETs)*,* identified four novel DNA methylation-defined brain tumor groups [19]. One group, termed high-grade neuroepithelial tumor with MN1 alteration (HGNET-MN1), showed recurrent rearrangements of the *MN1* gene, located at 22q12.3-qter. Reclustering of the DNA methylation data with tumors that did not carry a diagnosis of PNET revealed that approximately 40% of tumors clustering within the HGNET-MN1 DNA methylation class were institutionally diagnosed as AB [19]. Subsequent evaluation of limited cohorts confirmed that *MN1* rearrangements occur in a subset of ABs [10, 22].

The presence of *BRAF*^*V600E*^ mutations in some ABs and *MN1* rearrangements in others raises questions as to whether AB represents a distinct entity, or a histologic pattern exhibited by multiple glial tumor types. We, therefore, evaluated the molecular characteristics of 27 histologically-defined ABs using DNA methylation profiling, chromosomal copy number analysis, fluorescence in situ hybridization (FISH), and *BRAF* mutation analysis.

## Methods

### Cases

Construction of a clinical cohort of 28 AB cases was previously described [15]. The published cohort was augmented for this study with six additional histologically-defined ABs following appropriate Institutional Review Board approval. Seven cases from the original cohort were excluded due to insufficient material for further analysis. This left 27 cases in the current cohort. Patient demographics and pathologic data are presented in Table 1. Cases from our original cohort were designated with the corresponding case numbers from that study [15] and included cases C1, C3, C5–C14, C16, C17, C19–C24, and C26. New cases were designated C29–C34. Recurrent tumor from two cases (designated rC10 and rC33) was available for limited analysis; thus, there were 29 total samples from 27 unique patients.

**Table 1:**
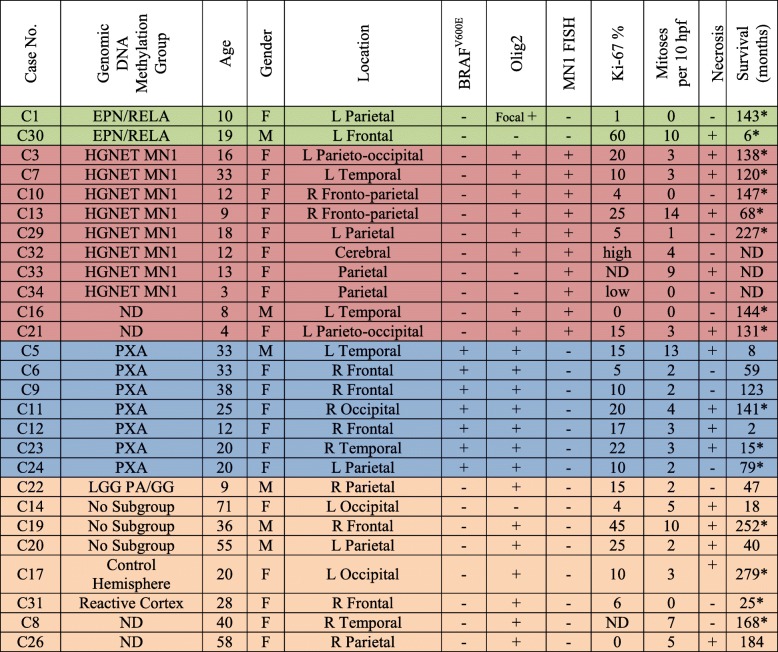
Astroblastoma patient demographics and pathology

Like or similar methylation groups are highlight by the same color

*Abbreviations*: *EPN/RELA* ependymoma with RELA fusion, *HGNET MN1* high-grade neuroepithelial tumor with MN1 alteration, *LGG PA/GG* low-grade glioma – supratentorial pilocytic astrocytoma/ganglioglioma, *ND* not determined due to inadequate tissue or unavailable follow-up data, *PXA* pleomorphic xanthoastrocytoma

*The patient was alive at most recent follow-up

### BRAF^V600E^ mutation analysis

Tumor *BRAF*^*V600E*^ mutation status was tested using a single nucleotide extension assay followed by Sanger sequencing as previously described [15]. *BRAF*^*V600E*^ testing of the new samples was performed as previously described [4].

### Immunohistochemistry

Immunohistochemical staining for olig2 and Ki-67 was performed as previously described [15].

### Fluorescence in situ hybridization

Dual-color FISH was performed on 4-μm paraffin-embedded tissue sections. Break-apart probes for *MN1* were derived from BAC clones RP11-432I9 and RP11-736H16 (BACPAC Resources, Oakland, CA). Probes were labeled with either AlexaFluor-488 (green) or AlexaFluor-555 (orange-red) fluorochromes (Invitrogen, Carlsbad, CA) and validated on normal control metaphase spreads. BAC probe mixtures were diluted 2:50 in hybridization buffer and co-denatured with the target cells on a slide moat at 90 °C for 12 min. The slides were incubated overnight at 37 °C on a slide moat followed by a 4 M Urea/2xSSC wash at 25 °C for 1 min. Nuclei were counterstained with DAPI (200 ng/ml) (Vector Labs, Burlingame, CA) for viewing with an Olympus BX51 fluorescence microscope equipped with a 100 watt mercury lamp; FITC, Rhodamine, and DAPI filters; 100X PlanApo (1.40) oil objective; and a Jai CV digital camera. Images were captured and processed with an exposure time ranging from 0.1 to 2 s for each fluorochrome using Cytovision v4.5 software (Leica Biosystems, Richmond, IL).

### DNA methylation array processing

DNA Extraction and bisulfite conversion of formalin-fixed, paraffin-embedded (FFPE) tissue sections was performed as previously described [5]. Briefly, DNA was extracted from FFPE tissue using the Maxwell 16 Plus LEV DNA purification kit (Promega, Madison, WI) according to the manufacturer’s instructions. Following bisulfite conversion with the Zymo, EZ DNA Methylation kit (Zymo Research, Irvine, CA), bisulfite DNA was processed using the Illumina Infinium HD FFPE Restore kit (Illumina, San Diego, CA) according to the manufacturer’s protocol. The DNA was then processed using the Illumina Infinium Methylation EPIC BeadChip kit (Illumina) and scanned on the Illumina HiScan system according to the manufacturer’s instructions. Beta values representing the fraction of methylated cytosine present at each CpG site were calculated with Illumina Genome Studio software using default settings. DNA methylation data analysis was performed with the statistical programming language R (R Core Team, 2016). Raw data files generated by the iScan array scanner were read and preprocessed using *minfi* Bioconductor package [2]. With the *minfi* package, the same preprocessing steps as in Illumina’s Genomestudio software were performed. In addition, the following filtering criteria were applied: removal of probes targeting the X and Y chromosomes; removal of probes containing-nucleotide polymorphism (dbSNP132 Common) within five base pairs of and including the targeted CpG-site; and removal of probes not mapping uniquely to the human reference genome (hg19), allowing for one mismatch. In total, 395,401 common probes of Illumina 450 K and EPIC arrays were kept for clustering analysis.

### Statistical analysis of DNA methylation

To determine the genomic DNA methylation pattern subgroup affiliation of our AB samples, we used the reference DNA methylation data published by Capper et al. available from the gene expression omnibus (GSE73801) [5]. Our AB samples were combined with the 2801 reference CNS tumors and control brain tissues for unsupervised hierarchical clustering as previously described [5]. In brief, the 32,000 most variable methylated CpG probes measured by standard deviation across combined samples were selected. 1-Pearson correlation was calculated as distance measured between samples and the unsupervised hierarchical clustering was performed by the average linkage agglomeration method. The probe level beta values were also analyzed using t-stochastic neighbor embedding (t-SNE) [20] with the *tsne* package (version 0.1–3) in R [7]. Hierarchical clustering and t-SNE analyses were repeated using a reduced reference set of tumors (*N* = 195) using the top 10,000 most differentially methylated probes. Supervised analysis was performed using the random forest methylation class prediction algorithm (V11b2) by uploading raw IDAT files to www.molecularneuropathology.org website [5].

### Detection of copy number aberrations

Copy number variation analysis from DNA methylation arrays was performed with the *conumee* Bioconductor package [11] using default settings. The combined intensities of all available CpG probes were normalized against control samples from normal brain tissue using a linear regression approach. Mean segment values of −0.18 and 0.18 were used as threshold to call copy number loss and gain, respectively. The control cohort used to evaluate the reference tumors from Capper et al. [5], profiled by 450 K DNA methylation analysis included all control samples from the dataset (*N* = 119). For copy number analysis of the AB samples, an alternative control cohort consisting of 26 normal brain samples profiled by the 850 K array was utilized.

### Kaplan-Meier analysis

Survival analysis was performed by Mantel-Cox log rank test with pairwise comparisons using IBM SPSS Statistics v. 19.0 software. A *P* value of 0.05 or less was considered statistically significant.

## Results

### Genomic DNA methylation

**Fig. 1:**
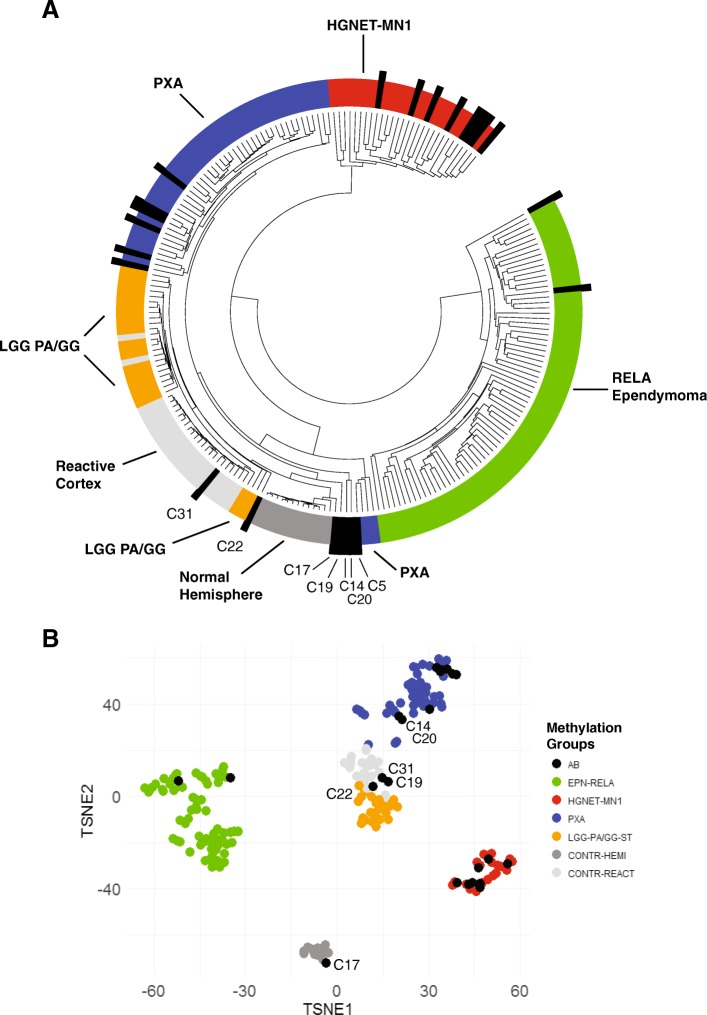
Histologically-defined ABs do not represent a homogeneous molecular group by DNA methylation profiling. The top 10,000 differentially methylated probes were used to perform unsupervised hierarchical clustering (**a**) or t-SNE analysis (**b**) of ABs and a reference group of tumors from selected DNA methylation classes from the Capper et al. dataset [5]

**Fig. 2:**
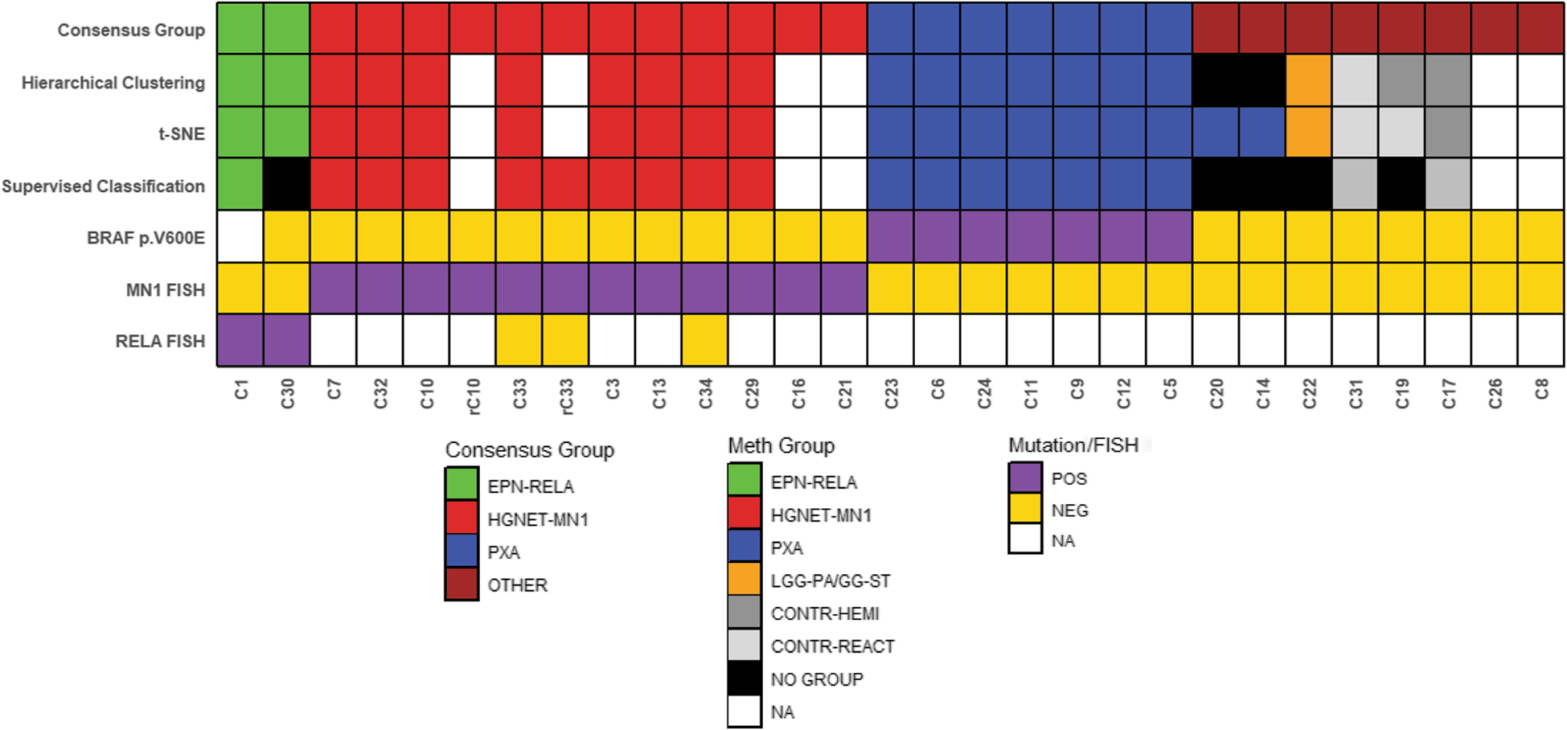
Orthogonal validation of molecular classification of ABs. Orthogonal testing including DNA methylation profiling, *BRAF*^*V600E*^ sequencing, and *MN1* and *RELA* FISH was performed on ABs with sufficient material. Supervised analysis was performed using the random forest methylation class prediction algorithm version 11b2 (www.molecularneuropathology.org). Recurrent tumors are indicated by “r”

850 K methylation analysis was performed on 23 of the 27 histologically-defined primary AB cases and 1 recurrent tumor (24 total samples). All but one sample (C21) yielded DNA methylation profiles of sufficient quality for subsequent analysis. Unsupervised hierarchical clustering and t-SNE analysis was performed by comparing the top differentially methylated probes first to a comprehensive reference series consisting of 2801 tumors representing all described DNA methylation classes (Additional file 1: Figure S1) [5]. This analysis was then reduced to a subset of 195 tumors consisting of: HGNET-MN1 (*n* = 21); PXA (*n* = 44); supratentorial ependymoma with *C11orf95-RELA* fusion (EPN-RELA; *n* = 70); supratentorial pilocytic astrocytoma and ganglioglioma (LGG-PA/GG-ST; *n* = 24); control reactive cortex (CONTR-REACT; *n* = 23); and control cerebral hemisphere (CONTR-HEMI; *n* = 13) (Fig. 1; Additional file 2: Figure S2). By unsupervised analysis, the ABs failed to cluster into a single group, and instead mostly distributed into previously defined DNA methylation classes. The results from hierarchical clustering and t-SNE analysis were concordant in 20 of 23 samples (Figs. 1 and 2). Eight tumors grouped with HGNET-MN1, seven with PXA, two with EPN-RELA, and one with LGG-PA/GG-ST. Two tumors clustered with either reactive cerebral cortex (C31) or control cerebral hemisphere (C17) (Fig. 1a). The latter was likely due to contamination from normal brain within the sample. An additional three tumors exhibited discordant grouping between the hierarchical clustering and t-SNE analysis. One of these (C19) was discordant between three methylation classes (CONTR-HEMI, CONTR-REACT, and LGG-PA/GG-ST). The other two tumors (C14 and C20) clustered together by hierarchical clustering, but not within any defined reference methylation class. They did, however, group with PXA by t-SNE analysis (Fig. 1b).

As an additional comparison, we also performed supervised analysis using the www.molecularneuropathology.org website, which employs a random forest methylation class prediction algorithm, using the comprehensive reference set used in the initial unsupervised clustering/t-SNE analysis [5]. For tumors with scores above the threshold values, the supervised analysis was concordant with the unsupervised methods (Fig. 2). Five tumors yielded a probability score below the reporting threshold of 0.90; however, in two of these (C22, and C30), the highest probability was consistent with the unsupervised hierarchical clustering analyses (LGG-PA/GG-ST 0.55 and EPEND RELA 0.89, respectively). Three additional cases (C14, C19, and C20) yielded unreliably low probability scores below 0.15 (Additional file 3: Table S1).

### Orthogonal validation of DNA methylation groups

Next, we performed orthogonal molecular analysis to determine if the ABs in specific DNA methylation classes contained the hallmark mutations or gene rearrangements of lesions within those classes. Limited molecular evaluation was also performed on tumors for which insufficient DNA was available for methylation analysis. All eight samples clustering with the HGNET-MN1 methylation class demonstrated evidence of *MN1* rearrangement by FISH (Fig. 2; Additional file 4: Figure S3). Support for an *MN1* rearrangement was also found by FISH in two additional cases for which insufficient material was available for DNA methylation analysis or for which methylation analysis failed (C16 and C21, respectively).

The *BRAF*^*V600E*^ mutation was identified in seven of nine tumors clustering in the PXA methylation group by t-SNE (Table 1). *BRAF*^*V600E*^ mutations were not detected in the two tumors in which t-SNE and hierarchical clustering were discordant (C14 and C20) (Figs. 1 and 2). The two tumors that clustered within the EPN-RELA methylation class were also found to have evidence of *RELA* rearrangement by interphase FISH.

### Chromosomal copy number

**Fig. 3:**
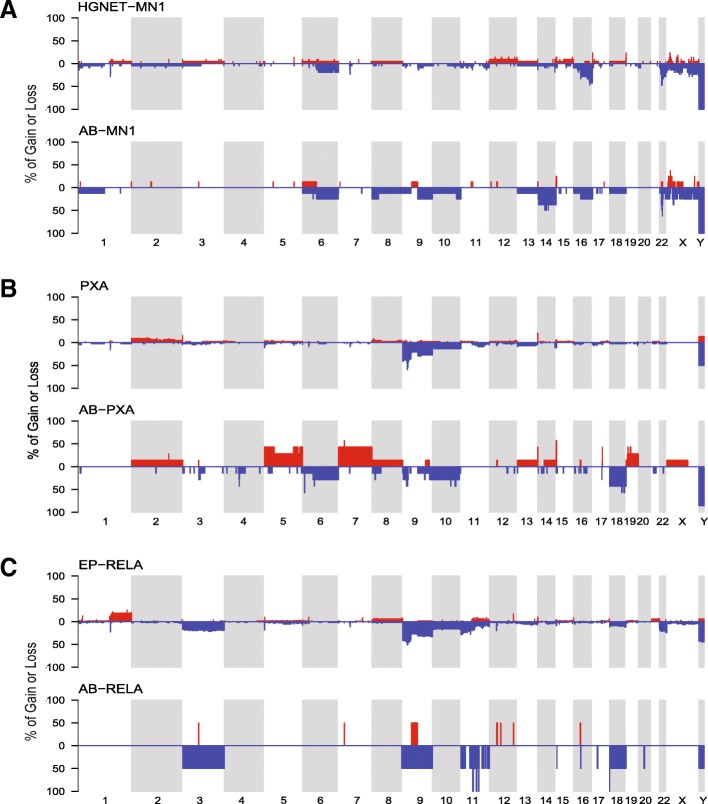
Chromosomal copy number variations in AB molecular groups compared to reference tumors. Copy number frequency plots were constructed using copy number profiles of reference tumors and ABs grouping with (**a**) HGNET-MN1, (**b**) PXA, and (**c**) EPN-RELA DNA methylation classes

To further evaluate the relationship between ABs and their respective molecular groups, we evaluated the copy number profiles of ABs compared to the respective reference tumors. ABs clustering in the HGNET-MN1 group variably demonstrated loss of chromosomes 22q, 14, and broad regions of X (three of eight cases), similar to previous findings reported in a small cohort of *MN1*-rearranged ABs [10] (Fig. 3a, Additional file 5: Figure S4A). These findings were consistent with those of the reference cohort of HGNET-MN1 tumors, with the exception of a slightly increased proportion of chromosome 14 loss in ABs.

*BRAF*^*V600E*^-positive ABs that grouped with PXA showed more extensive chromosomal instability compared to other ABs (Fig. 3; Additional file 5: Figure S4A), with frequent gains of chromosome 5, chromosome 7, and chromosome 19; and loss of chromosomes 10, 18, and 6q. Unlike commonly observed in glioblastoma and in the approximately 20% of PXAs (especially anaplastic PXAs) with chromosome 7 and 10 aberrations [21], gain of chromosome 7 and loss of chromosome 10 were mutually exclusive in ABs (Additional file 5: Figure S4A). While interpretation of data for ABs clustering with EPN-RELA was limited by case number, observed copy number variations were compatible with findings in the reference cohort of RELA ependymomas (Fig. 3c).

Tumors showed few recurrent focal copy number abnormalities within or between DNA methylation classes. Loss of the *CDKN2A/B* locus was observed in one tumor each grouping with HGNET-MN1 and EPN-RELA (Additional file 5: Figure S4B). Three of seven tumors that both grouped with PXA and contained *BRAF*^*V600E*^ mutations showed focal copy number loss at *CDKN2A/B* (Additional file 5: Figure S4B). Neither *BRAF* wildtype tumors grouping with PXA in the t-SNE analysis (C14 and C20) showed *CDKN2A/B* loss, nor did any of the other tumors lacking known driver mutations. No other recurrent focal copy number abnormalities were observed across any of the groups.

### Histopathology

**Fig. 4:**
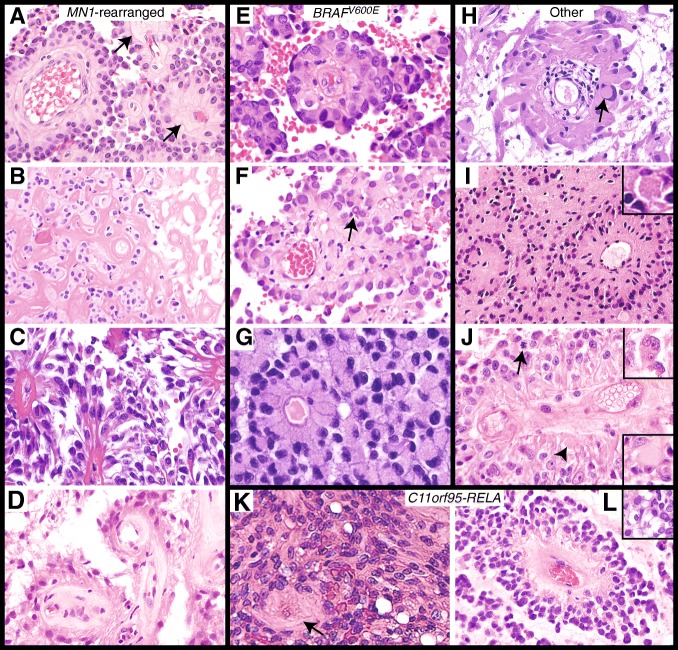
AB tumor histology. **a**–**d** *MN1*-rearranged tumors: (**a**) Case C10 showing mild vascular sclerosis (arrows). This image depicts recurrent tumor. **b** Case 10. The original lesion was highly sclerotic. **c** Case C3 showed thin tapering process with expanded endfeet in some areas (shown) and stouter clear cells in other areas. Mild vascular sclerosis is again noted. **d** Case C16 demonstrating fibrillary areas and vascular sclerosis. **e**–**g** *BRAF*^*V600E*^-positive cases: (**e**) Case C12 with mild vascular sclerosis. **f** Case C11. A pseudonuclear inclusion is present (arrow). **g** Case C6. **h**–**j** Cases without known driver mutations: (**h**) Case 22 showing monopolar columnar-like cells and a pseudonuclear inclusion (arrow). **i** Case C14. An eosinophilic granular body is shown in the inset. **j** Case C19 demonstrating mitotic activity (arrow), free hyaline bodies (arrowhead), intracellular hyaline bodies (lower inset), and multinucleate cells (upper inset). **k**–**l** *C11orf95-RELA* tumors: (**k**) Case C1, low-grade *C11orf95-RELA* lesion showing AB histology and mild vascular sclerosis (arrow). **l** Case C30 demonstrating ependymal-like pseudorosettes and clear signet ring-like cells (inset)

*MN1-*rearranged, *BRAF*^*V600E*^-mutant, *RELA-*rearranged, and tumors without identified driver mutations all occasionally demonstrated nuclear pseudoinclusions [15] (Fig. 4f and h). *MN1-*rearranged tumors more often showed vascular and/or generalized sclerosis. Three of the 10 *MN1-*rearranged tumors demonstrated marked sclerosis and contained hyalinized areas consisting of nearly entirely sclerotic vessels as depicted to the right in Fig. 4b. Although the most marked sclerosis was seen in this group, mild vascular sclerosis was also occasionally seen in tumors in the other molecular groups (Fig. 4e, j, and k). *BRAF*^*V600E*^-mutant ABs tended to have stouter cells (Fig. 4e-g); however, such cells were also occasionally seen in *MN1-*rearranged and other tumors (Fig. 4a).

When examining AB histological features which we previously cataloged [15], *RELA*-rearranged tumors often showed clear or signet ring-like cells (Fig. 4k and l); however, these were also observed in select tumors from the other types. Eosinophilic granular body–like structures and spheroid hyaline bodies were absent in *RELA-* and *MN1*-rearranged tumors, but were common in *BRAF*^*V600E*^*-*mutant tumors and tumors without known driver mutations (Fig. 4i and j). Rhabdoid-like cells were not identified in *RELA-*rearranged tumors, but were found in all other types. All but two *MN1*-rearranged tumors and both *RELA*-rearranged tumors lacked lymphocytic infiltrates, which were common in other types. Multinucleated cells were present in almost all tumors except two *MN1*-rearranged tumors, the low-grade *RELA-*rearranged lesion (C1) and one other lesion (C2). Overall, *RELA-*rearranged tumors tended to lack some histologic features common in most other groups; e.g., lymphocytic infiltrates, eosinophilic granular material, and rhabdoid-like cells.

### Clinicopathologic correlates

Nine of the 10 consensus tumors with *MN1* rearrangement (90%) presented in female patients aged 3–33 years and 1 was from an 8-year-old male (mean age, 12.8 years; median, 12 years) (Table 1). Eight of 10 *MN1-*rearranged tumors were olig2 immunopositive, and none showed hypermethylation of the O^6^-methylguanine methyl transferase (*MGMT)* gene promoter via the EPIC BeadChip (Table 1). Similarly, six of seven *BRAF*^*V600E*^-mutant ABs (87.5%) occurred in females aged 12–38 years and one presented in a 33-year-old male patient (mean, 25.9 years; median, 25 years) (Table 1). All were olig2 immunopositive. Two cases (C5 and C9), from patients 33 and 38 years-of-age, respectively, exhibited *MGMT* promoter hypermethylation, as previously described [15].

*RELA* rearrangements were detected in tumors from one female and one male (C1 and C30), aged 10 and 19 years, respectively. Olig2 immunohistochemical staining was equivocal in C1 and negative in C30. Neither showed *MGMT* promoter hypermethylation. Six other tumors negative for *BRAF*^*V600E*^ mutations and *MN1 or RELA* rearrangements consisted of lesions from three females and three male patients ranging in age from 4 to 71 years. None showed *MGMT* promoter hypermethylation. All were olig2 immunopositive, except C14 [15].

### Survival analysis

**Fig. 5:**
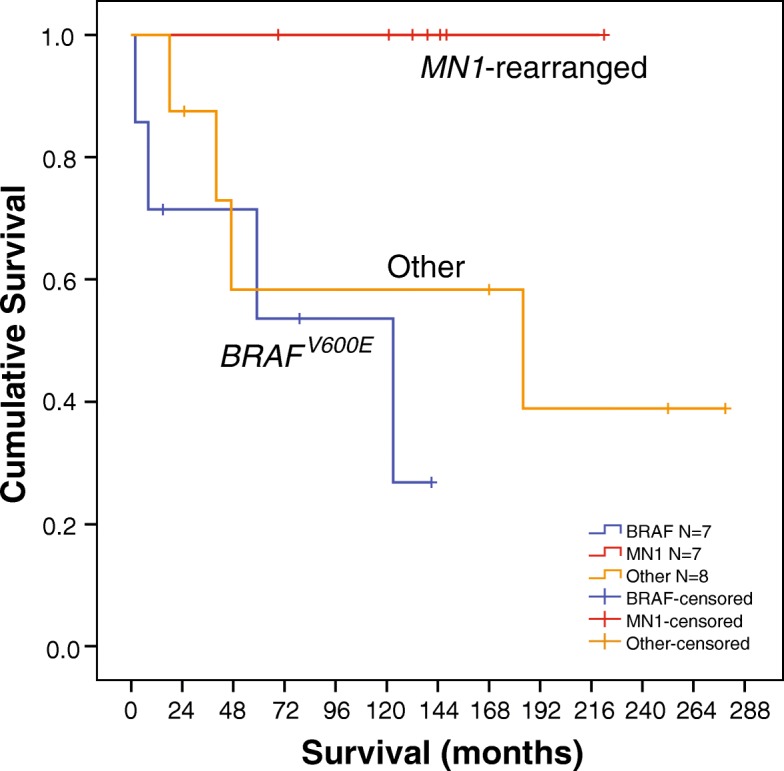
*MN1*-rearranged ABs show significantly better survival compared to *BRAF*^*V600E*^-mutant ABs. The initial numbers of patients in each group are indicated in the key. The two RELA/EPEN tumors were not included in the analysis

Survival analysis was statistically limited by the relatively small number of tumors in each group, but overall showed a significant difference between molecular groups (Mantel-Cox, *P* = 0.045; Fig. 5). In pairwise analysis, there was no appreciable difference in overall survival between ABs with *BRAF*^*V600E*^ mutations and tumors without specific driver mutations (*P* = 0.398). There did appear to be a difference in survival between *MN1*-rearranged tumors and tumors without identified driver mutations (other tumors); however, this did not reach statistical significance (*P* = 0.056). There was, however, a clear and significant survival advantage for *MN1*-rearranged tumors compared to *BRAF*^*V600E*^-mutant tumors (*P* = 0.013; Fig. 5). In fact, all *MN1-*rearranged tumor patients in the cohort are currently alive, despite multiple tumor recurrences in some cases (Additional file 6: Table S2). Four deaths each occurred in the seven *BRAF*^*V600E*^ mutation patients and in the eight patients without known driver mutations (Table 1). The overall survival of the *MN1*-rearranged tumor patients’ ranged from 68 to 221 months (mean, 138 months; *n* = 7) compared to 2 to 141 months (mean, 61 months; *n* = 7) for patients whose tumors had *BRAF*^*V600E*^ mutation, and 18 to 279 months (mean, 127 months; *n* = 8) for patients with neither genetic alteration.

## Discussion

Studies of limited cohorts have suggested genetic heterogeneity among AB cases [3, 22]; whereas, others have argued that AB is a distinct entity characterized by *MN1* rearrangement [10]. Our findings confirm that histologically-defined ABs do not represent a single molecular tumor entity, but instead largely cluster into two known genomic DNA methylation classes: HGNET-MN1 and PXA.

Tumors designated HGNET-MN1 were previously identified in a cohort of PNETs [19]; however, whether tumors with that designation should be treated as embryonal tumors remains controversial. ABs with *MN1* rearrangement presented herein were notable for favorable overall survival. Importantly, they did not demonstrate embryonal features, and were not uniformly high grade, but instead showed a mixture of low- and higher-grade histologies reflected by degree of mitotic activity and the absence or presence of necrosis. These findings suggest that, despite initial discovery in a cohort of tumors diagnosed as PNET, the relationship between histomorphology and clinical behavior in *MN1*-rearranged tumors requires further evaluation. Given the relatively good overall survival associated with these lesions, a more conservative therapeutic approach may suffice.

The second major molecular group in our AB cohort included tumors grouping with PXA by DNA methylation. While especially *BRAF*^*V600E*^-mutant ABs showed genomic methylation patterns and other genetic changes common to PXA (e.g., *CDKN2A/B* deletion in three cases), several findings suggest they may not be entirely equivalent entities. For instance, PXAs show a stronger predilection for the temporal lobe, and occur in equal frequency in males and females [6, 12]. It is, therefore, likely that the PXA reference DNA methylation group is relatively heterogenous compared to other methylation classes and encompasses tumors other than conventional PXAs. This is supported by studies suggesting that other *BRAF*^*V600E*^-mutant tumors, such as epithelioid glioblastoma, molecularly group with PXA [13].

A small percentage of ABs grouped with RELA ependymomas by genomic DNA methylation analysis. The latter tumors [18] are generally associated with a poorer prognosis [17] and should be distinguished from other AB-like lesions. Thus, AB-like tumors should be investigated for *RELA* fusion by FISH or screened by immunohistochemistry for p65/RELA and/or L1CAM [8]. RELA ependymomas can be further differentiated from other AB pseudorosette-predominant lesions by *BRAF* mutational analysis, FISH for *MN1* rearrangement, or genomic DNA methylation analysis [5].

Previous studies have variably argued that some ABs are related to diffuse astrocytomas. Our data do not support that assertion as we did not identify ABs that molecularly grouped with diffuse astrocytomas. This is likely due to such studies not applying our relatively strict criteria for histopathologic designation of AB including: requiring that an AB case demonstrate at least 50% AB pseudorosettes and show relative tumor circumscription without evidence of an invasive growth pattern.

DNA methylation profiling is a powerful tool for tumor classification that can overcome shortcomings of histopathology and more conventional molecular testing, allowing for accurate classification of histologically ambiguous tumors [5, 19]. However, we also found tumors that clustered with no known reference DNA methylation class. Furthermore, Capper et al. [5] described a tumor histologically resembling AB as unclassifiable by DNA methylation profiling. These findings suggest that additional drivers, other than *MN1* rearrangements, *BRAF*^*V600E*^ mutations, and *RELA* fusions, may exist for tumors with AB histology. Expansion of existing tumor methylation reference sets may therefore be necessary to allow classification of such tumors by DNA methylation profiling.

## Conclusions

Regardless of molecular heterogeneity, AB remains a recognizable histological pattern reflecting tumors with important prognostic and treatment implications, notably their amenability to surgical resection and an overall better prognosis compared to diffuse gliomas. Although survival analysis between molecularly-defined AB subtypes was limited by sample size, tumors with *MN1* rearrangement were characterized by a statistically significant, more favorable outcome compared to *BRAF*^*V600E*^-mutant ABs, emphasizing the importance of recognizing AB molecular subtypes for their prognostic and possible treatment implications.

## Additional files

Additional file 1: **Figure S1.** Unsupervised t-SNE analysis of ABs with the entire Capper et al. dataset [5]. DNA methylation profiles from ABs were analyzed with the comprehensive Capper et al. dataset using the top 32,000 probes. (**A**). ABs did not group into a single methylation class, but instead grouped within the methylation classes designated (**B**) EPN-RELA, (**C**) PXA, (**D**) HGNET-MN1, (**E**) CONTR-REACT and LGG-PA/ST-GG, and (**F**) CONTR-HEMI. (EPS 5516 kb)

Additional file 2: **Figure S2.** Heatmap from unsupervised hierarchical clustering of ABs with selected reference tumors from the Capper et al. dataset [5] using the top 10,000 differentially methylated probes. (PDF 94854 kb)

Additional file 3: **Table S1.** Tumor DNA methylation class probabilities from the random forest methylation class prediction algorithm. The lowest probabilities are shaded in blue and the highest probabilities are shaded in red. (XLSX 42 kb)

Additional file 4: **Figure S3.** Example images of *MN1* FISH. (**A**) Case C10 showing red and green break apart probes (arrows) and yellow intact *MN1* loci (arrowheads). (**B**) Case C3 with displaced green probe only indicating an unbalanced translocation (arrows). (**C**) Case C23 showing intact *MN1* loci. (**D**) Case C11 showing multiple intact *MN1* loci per nucleus indicating chromosome 22 polyploidy. (EPS 23150 kb)

Additional file 5: **Figure S4.** Chromosome copy number analysis. (**A**) Copy number analysis of individual AB samples. (**B**) Expanded view of chromosome 9. The consensus DNA methylation groups are annotated by EPN-RELA = “green”, HGNET-MN1 = “red”, PXA = “blue”, and other/unknown = “brown”. Recurrent tumor is indicated by “r”. (PDF 1754 kb)

Additional file 6: **Table S2.** Patient Treatment and Survival Data. (DOCX 25 kb)
