# Supplementary figures and images for: Genomic analysis demonstrates that histologically-defined astroblastomas are molecularly heterogeneous and that tumors with MN1 rearrangement exhibit the most favorable prognosis
Source: Acta Neuropathol Commun. 2019 Mar 15;7:42. doi: 10.1186/s40478-019-0689-3 (PMC6419470; doi:10.1186/s40478-019-0689-3)

- AB
- EPN-RELA
- HGNET-MN1
- LGG-PA/GG-ST
- PXA
- CONTR-HEMI
- CONTR-REACT

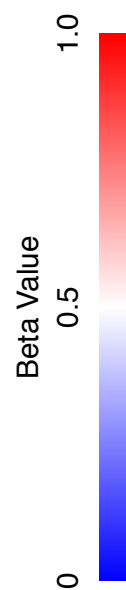

DNA methylation probes (n=10,000)

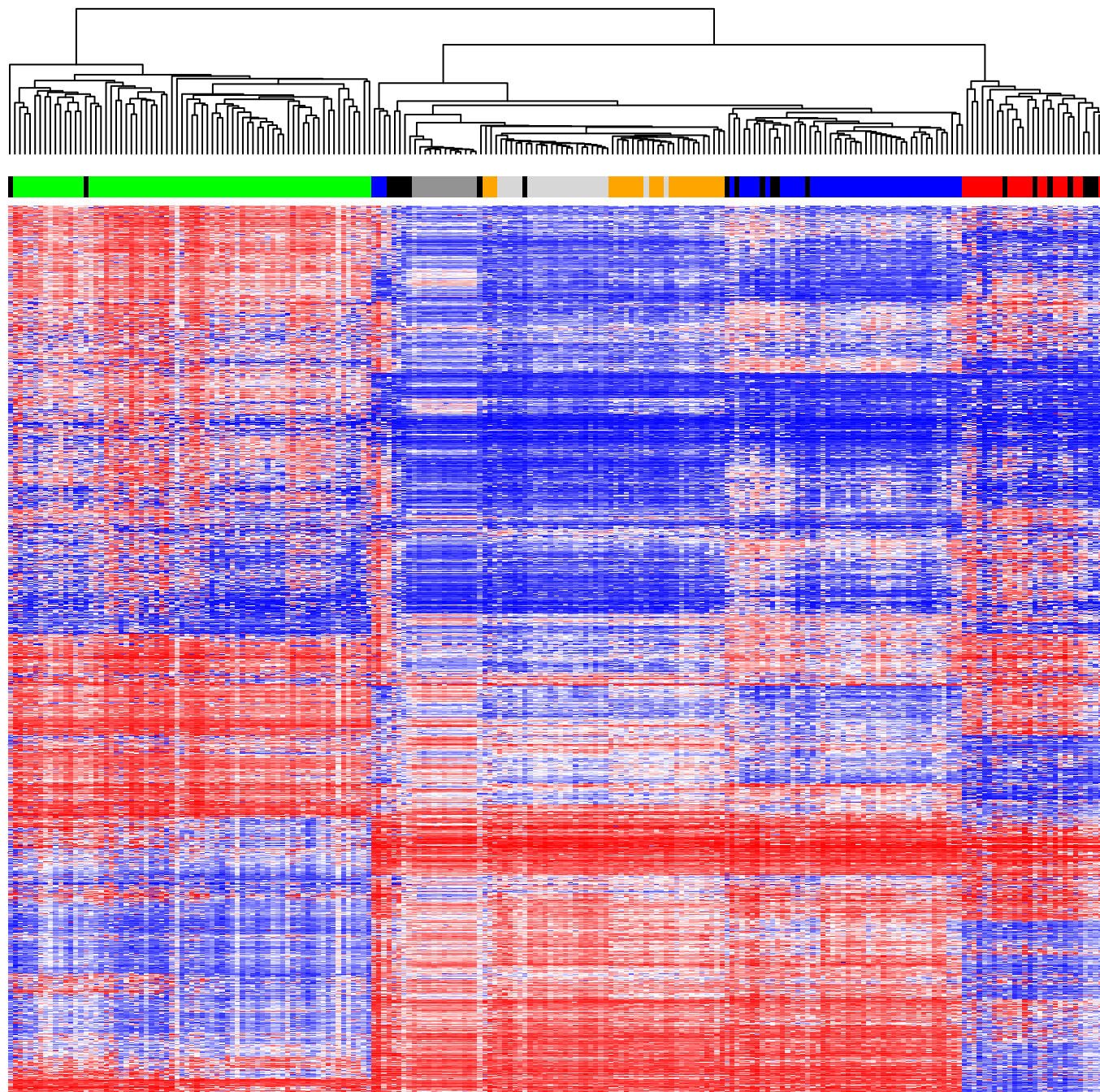

Supplement: Supplementary file 2 — Figure S2. Heatmap from unsupervised hierarchical clustering of ABs with selected reference tumors from the Capper et al. dataset [5] using the top 10,000 differentially methylated probes. (PDF 94854 kb) [file 40478_2019_689_MOESM2_ESM.pdf]

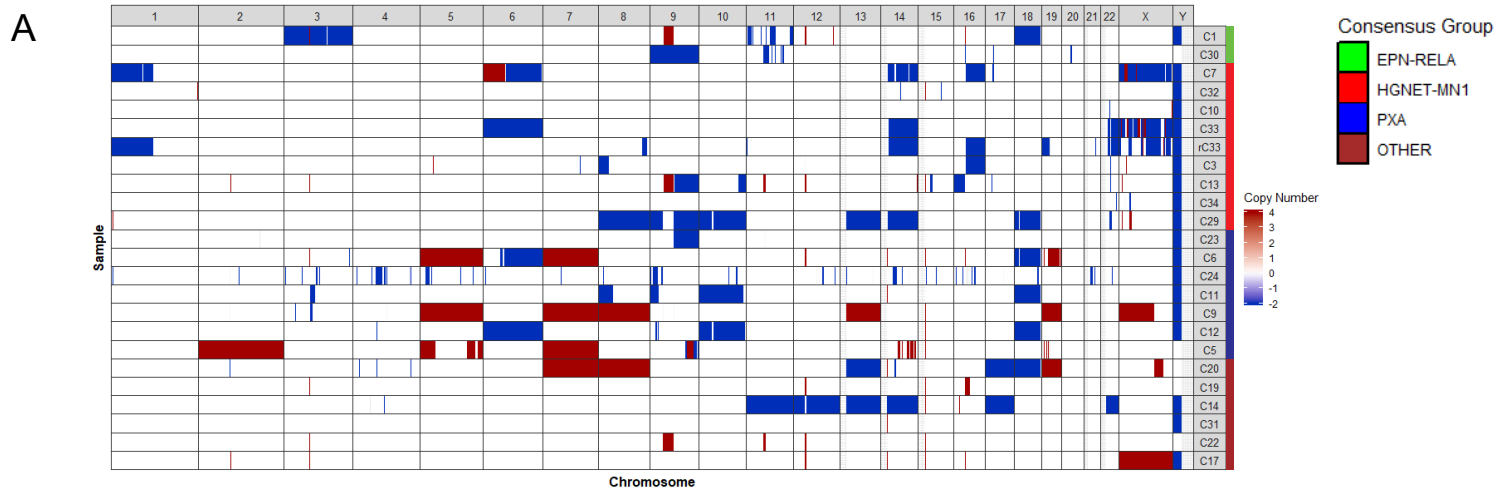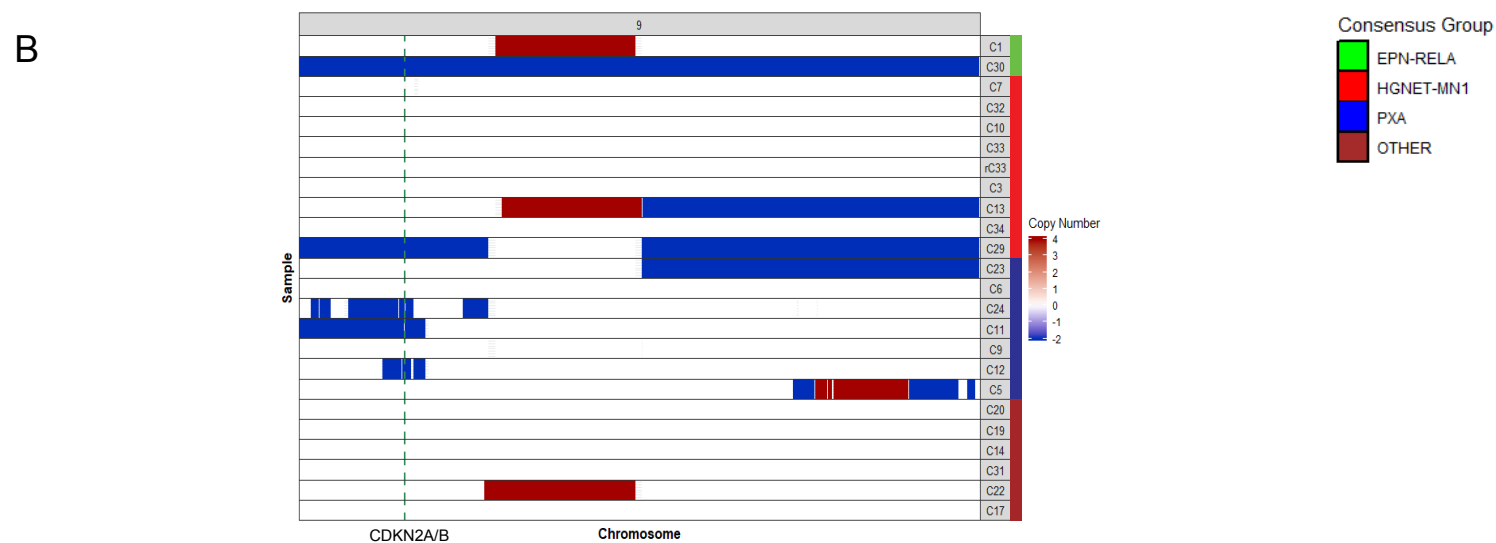

Supplement: Supplementary file 5 — Figure S4. Chromosome copy number analysis. (A) Copy number analysis of individual AB samples. (B) Expanded view of chromosome 9. The consensus DNA methylation groups are annotated by EPN-RELA = “green”, HGNET-MN1 = “red”, PXA = “blue”, and other/unknown = “brown”. Recurrent tumor is indicated by “r”. (PDF 1754 kb) [file 40478_2019_689_MOESM5_ESM.pdf]
