# Supplementary material for: Genomic analysis demonstrates that histologically-defined astroblastomas are molecularly heterogeneous and that tumors with MN1 rearrangement exhibit the most favorable prognosis
Source: Acta Neuropathol Commun. 2019 Mar 15;7:42. doi: 10.1186/s40478-019-0689-3 (PMC6419470; doi:10.1186/s40478-019-0689-3)
Supplement: Supplementary file 6 — Table S2. Patient Treatment and Survival Data. (DOCX 25 kb) [file 40478_2019_689_MOESM6_ESM.docx]

Table S2. Survival Data

| **Case** | **Age**  **(years)**  **Sex** | **Recurrence**  **Additional Treatment** | **Survival in months** |
| --- | --- | --- | --- |
| C1 | 10F | none | 143* |
| C2 | 22M | Multiple local recurrences at approx. 3, 6, 8 yrs. RT to 4^th^ ventricle at 4 years, chemo for 3 mo. at 5 years | 186* |
| C3 | 16F | Multiple local recurrences at approx. 1, 4, 5, 8 and 11 years | 138* |
| C5 | 33M |  | 8 |
| C6 | 33F | deceased | 59 |
| C7 | 33F |  | 120* |
| C8 | 40F |  | 168* |
| C9 | 38F | Recurred at 7 months, deceased | 123 |
| C10 | 12F | Recurred at approx. 4.3 years | 147* |
| C11 | 25F | At 4 months, after completion of RT, MRI showed 8mm focus of residual tumor; underwent a second resection at 5 months | 141* |
| C12 | 12F | deceased | 2 |
| C13 | 9F |  | 68* |
| C14 | 71F | deceased | 18 |
| C16 | 8M |  | 144* |
| C17 | 20F | Recurred at approx. 3 years | 279* |
| C19 | 36M | GTR, external beam RT, 59.4 Gy, stereotactic boost 8 Gy, then BCNU 6 cycles, pseudo-progression radiation necrosis 12/98, biopsy 1/99, biopsy early 2000, biopsy 6/22/2001 | 252* |
| C20 | 55M | deceased | 40 |
| C21 | 4F |  | 131* |
| C22 | 9M | Recurred at 1.5 years,  deceased | 47 |
| C23 | 20F | Received proton beam RT with concurrent temozolomide at 2 months to 3.25 months after GTR, then monthly temozolomide at 200 mg/m2.  Completed 12th cycle at 1.25 years, Stable MRI | 15* |
| C24 | 20F | No known recurrence | 61* |
| C25 | 14M | Recurred at 12 months,  deceased | 39 |
| C26 | 58F | Recurred at 12 years, deceased 3.5 years later | 184 |
| C29 | 18F | Recurred after GTR at approx. 2, 4, 4.5 years, 4.5 RT | 227* |
| C31 | 28F | none | 25* |

Recurrences were treated with resection unless otherwise noted.

Abbreviations: RT, radiation therapy; * The patient is still alive.

No clinical follow-up is available for cases C32-34.
